# Characteristics of persistent hotspots of *Schistosoma mansoni* in western Côte d’Ivoire

**DOI:** 10.1186/s13071-020-04188-x

**Published:** 2020-07-02

**Authors:** Rufin K. Assaré, Roméo N. N’Tamon, Louise G. Bellai, Judicaelle A. Koffi, Tra-Bi I. Mathieu, Mamadou Ouattara, Eveline Hürlimann, Jean T. Coulibaly, Salia Diabaté, Eliézer K. N’Goran, Jürg Utzinger

**Affiliations:** 1 Unité de Formation et de Recherche Biosciences, Université Félix Houphouët-Boigny, 22 BP 582, Abidjan 22, Côte d’Ivoire; 2 Centre Suisse de Recherches Scientifiques en Côte d’Ivoire, 01 BP 1303, Abidjan 01, Côte d’Ivoire; 3 Swiss Tropical and Public Health Institute, CH-4002, Basel, Switzerland; 4 University of Basel, CH-4003, Basel, Switzerland; 5 Unité de Formation et de Recherche Science de l’Homme et de la Société, Université Félix Houphouët-Boigny, 08 BP 865, Abidjan 08, Côte d’Ivoire; 6 Centre d’Entomologie Médicale et Vétérinaire, 27 BP 529, Abidjan 27, Côte d’Ivoire

**Keywords:** *Biomphalaria pfeifferi*, Côte d’Ivoire, Persistent hotspot, Preventive chemotherapy, *Schistosoma mansoni*, Schistosomiasis, Water, sanitation and hygiene (WASH)

## Abstract

**Background:**

Preventive chemotherapy with praziquantel is the cornerstone of schistosomiasis control. However, in some social-ecological settings, the prevalence and/or intensity of *Schistosoma* infection does not lower meaningfully despite multiple rounds of preventive chemotherapy, a phenomenon termed persistent hotspot (PHS). We assessed the characteristics of PHS in a *Schistosoma mansoni*-endemic area of Côte d’Ivoire.

**Methods:**

In October 2016, a cross-sectional survey was conducted in 14 schools in the western part of Côte d’Ivoire, one year after multiple rounds of preventive chemotherapy. In each school, 50 children aged 9–12 years provided two stool samples and one urine sample. Stool samples were subjected to triplicate Kato-Katz thick smears for *S. mansoni* diagnosis. Urine samples were examined by a filtration method for *S. haematobium* eggs. PHS was defined as failure to achieve a reduction in the prevalence of *S. mansoni* infection of at least 35% and/or a reduction of infection intensity of at least 50%. Six schools underwent more detailed investigations, including a questionnaire survey for demographic characteristics and a malacological survey.

**Results:**

In the six schools subjected to detailed investigations, the overall prevalence of *S. mansoni* and *S. haematobium* was 9.5% and 2.6%, respectively. Four schools were classified as PHS. The *S. mansoni* prevalence in the four PHS was 10.9% compared to 6.6% in the remaining two schools. The *S. mansoni* infection intensity, expressed as arithmetic mean eggs per gram of stool (EPG) among infected children, was 123.8 EPG in PHS and 18.7 EPG in the other two schools. Children bathing in open freshwater bodies were at higher odds of *S. mansoni* infection (odds ratio: 4.5, 95% confidence interval: 1.6–12.6). A total of 76 human-water contact sites (53 in PHS and 23 in the other schools) were examined and 688 snails were collected, including potential intermediate host snails of *Schistosoma* (*Biomphalaria pfeifferi*, *Bulinus forskalii*, *Bu. globosus* and *Bu. truncatus*).

**Conclusion:**

Children in PHS schools bathed more frequently in open freshwater bodies, and hence, they are more exposed to *Schistosoma* transmission. Our findings call for an integrated control approach, complementing preventive chemotherapy with other interventions, particularly in PHS settings.
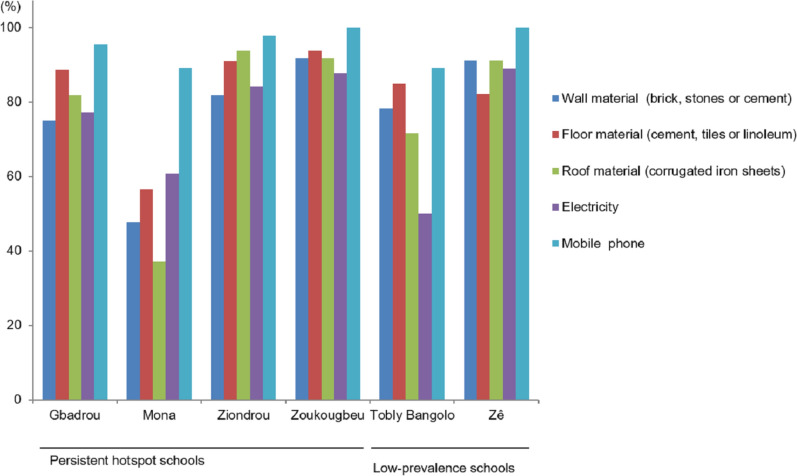

## Background

Although schistosomiasis affects more than 250 million people, it is considered a neglected tropical disease that caused an estimated burden of 1.4 million disability-adjusted life years in 2017 [1, 2]. Preventive chemotherapy with praziquantel is the mainstay of the global schistosomiasis control strategy [3]. In 2016, 52.8 million school-aged children requiring preventive chemotherapy received praziquantel, a coverage of 54% [4].

In order to strengthen the current evidence-base for decisions about preventive chemotherapy for gaining and sustaining control of schistosomiasis, the Schistosomiasis Consortium for Operational Research and Evaluation (SCORE) implemented a series of large-scale, multi-year trials in sub-Saharan Africa [5, 6]. In Côte d’Ivoire, a SCORE study pertaining to sustaining control of schistosomiasis mansoni was carried out in 75 schools in the western part of the country. Schools were included when the prevalence of *Schistosoma mansoni* was between 10% and 24%, as determined by duplicate Kato-Katz thick smears among 50 children aged 13–14 years [7]. The selected schools were randomly allocated to one of three treatment arms, including 25 schools per arm. Children in arm A received annual praziquantel for four years, children in arm B were administered praziquantel in the first two years, followed by two years of “drug holidays”, while children in arm C received treatment in years 1 and 3, alternated by “drug holidays” in years 2 and 4. The dynamics of schistosomiasis was heterogeneous, as revealed by the initial treatment follow-up one year after the first drug administration. Indeed, while in 43 of the 50 schools surveyed at this time point, a decrease in *S. mansoni* prevalence was observed, in seven schools, the prevalence increased [8]. Similar results have been reported elsewhere in sub-Saharan Africa [9–12].

Social-ecological settings where the prevalence and/or intensity of *S. mansoni* increases or does not change meaningfully after preventive chemotherapy are termed persistent hotspots (PHS). There are different definitions of PHS. The approach adopted in the current study has been described elsewhere [13]. In brief, a village that failed to reduce the *S. mansoni* infection prevalence by at least 35% and/or failed to reduce the intensity of infection by at least 50% between baseline and year five testing is considered a PHS, while a village that fulfils these criteria is termed a low-prevalence setting. Of note, PHS of schistosomiasis have been investigated in different parts of the world [14–17] and in the gaining and sustaining control studies of SCORE [13]. Prior research showed that various factors can contribute to the existence of PHS, including close proximity of human habitation to open surface water bodies where intermediate host snails proliferate, human behaviours, particularly in terms of water, sanitation and hygiene (WASH) parameters [18–20] and socioeconomic status of the study population [21, 22].

In the western part of Côte d’Ivoire where a multi-year sustaining schistosomiasis mansoni control study was conducted, 45% (34 of 75) of the investigated villages were PHS [13]. However, the factors that drive PHS in this area are unknown. Hence, new research is needed to investigate factors, including human behaviours and environmental characteristics that might explain persistence of pockets of schistosomiasis transmission [13, 15, 17]. The specific objectives of this study were to: (i) characterise PHS of *S. mansoni* in a SCORE sustaining control study in western Côte d’Ivoire; (ii) determine environmental and demographic factors and WASH indicators that might govern PHS; (iii) assess the awareness of the study population on schistosomiasis and praziquantel treatment; and (iv) identify intermediate host snails and determine whether they shed schistosome cercariae.

## Methods

### Study area and population

The study was carried out between October and December 2016 in Cavally, Guemon, and Haut-Sassandra, three regions of western Côte d’Ivoire (geographical coordinates: 06°32′42.0″ to 07°36′54.8″N latitude; 06°44′09.8″ to 07°33′48.9″W longitude) [7, 23]. The villages of Gbadrou, Tobly Bangolo, Zê and Ziondrou are located in the Guemon region, while Mona and Zoukougbeu belong to the Cavally and Haut-Sassandra region, respectively. The hydrological system is dominated by the Sassandra River and two tributaries; the N’zo River on the right bank and the Lobo River on the left bank [24]. The regions of Cavally and Guemon are situated West of the Sassandra River and belong to the district “des Montagnes”, which is a mountainous area with an average altitude ranging between 300 m and slightly above 1000 m above sea level. The climate is humid tropical with two seasons. The rainy season lasts from March to October. The Haut-Sassandra region is located East of the Sassandra River and belongs to Sassandra-Marahoué district. Zoukougbeu is a town located in Sassandra-Marahoué district. The average altitude ranges between 200 m and 300 m above sea level. The climate is sub-equatorial, characterised by two rainy seasons with the long rainy season occurring from March to July and the short rainy season in September and October.

In 2014, there were 1000, 2400, 10,550, 1040, 840 and 11,860 inhabitants in Gbadrou, Mona, Tobly Bangolo, Zê, Ziondrou and Zoukougbeu, respectively (unpublished data; Institut National de la Statistique en Côte d’Ivoire). People in these villages belong to three main ethnic groups: Bété, Guéré and Wobé. The two main religions are Christianism and Islam, while a considerable proportion of people are animists. Most people are engaged in subsidence farming (e.g. cassava, maize, plantain and rice). The two main cash crops are coffee and cocoa. Fishing is also observed, mainly by allochthonous in the man-made Buyo lake. The annual rainfall in the study area varies between 1100 and 2000 mm. The vegetation is composed of two types of forests (semi-deciduous and evergreen mountain forest). The average annual temperature is around 26 °C.

Preventive chemotherapy targeting school-aged children was conducted in the study areas in late 2015 [7]. Infection with *S. mansoni* is highly endemic in this part of Côte d’Ivoire [23, 25, 26]. A longitudinal malacological survey carried out monthly from 1986 to 1988 in two villages in the Tonkpi region revealed the presence of *Biomphalaria pfeifferi*, the intermediate host snail of *S. mansoni* [27].

In the frame of a large SCORE-funded project, a parasitological survey was conducted in 2011 and 2012, in 75 purposely selected communities to determine the baseline situation and village characteristics before the implementation of a 4-year randomised controlled trial designed to measure the impact of three different school-based treatment schedules with praziquantel to sustain the control of *S. mansoni* infection [7, 28]. There was an overall prevalence of *S. mansoni* of 5.4% among first-graders and 22.1% in children aged 9–12 years [28]. One year after the initial administration of praziquantel, the overall prevalence of *S. mansoni* in children aged 9–12 years in 50 schools decreased significantly from 19.7% (95% confidence interval (CI): 18.5–20.8%) at baseline to 12.8% (95% CI: 11.9–13.8%) at the 1-year follow-up with considerable spatial heterogeneity in the dynamic of *S. mansoni* infection. While the prevalence of *S. mansoni* decreased in most of the schools, in some schools the prevalence remained unchanged or even increased.

In October 2016, a sub-study was launched in 14 schools, consisting of a parasitological survey in all 14 schools [29], and a malacological and questionnaire survey in six of the schools. Limited financial resources, challenges in accessing some of the schools and other logistic reasons precluded surveying all 14 schools. Among the six schools, three had a *S. mansoni* prevalence above 24%, and hence, were considered highly endemic according to a pre-set SCORE threshold. These three villages are Ziondrou (32.7%), Zê (27.0%) and Tobly Bangolo (26.0%). The other three schools were classified as moderately endemic (10–24% prevalence): Gbadrou (19.0%), Zoukougbeu (15.2%) and Mona (14.0%).

### Parasitological survey

In a first step, a cross-sectional parasitological survey was conducted in the 14 schools. In brief, in each school, 50 children aged 9–12 years were randomly selected and invited to participate. We selected 50 children per school, according to guidelines put forth by the World Health Organization (WHO) [30]. Children aged 9–12 years were chosen in accordance to SCORE protocols and the fact that children at this age are particularly active, frequently contact open freshwater bodies and are thus exposed to schistosomiasis [30, 31]. On the first day of the survey, children with written parental informed consent received two empty plastic containers. They were asked to fill one container with a small portion of fresh morning stool and to collect a mid-day urine sample in the second container. On the second day, children provided an additional stool sample but no further urine sample. All stool samples were subjected to triplicate 41.7 mg Kato-Katz thick smears [32]. After a clearing time of at least 30 min, the thick smears were examined under a microscope by one of four experienced laboratory technicians. Urine samples were examined using a filtration method [33]. Eggs of *S. mansoni* and *S. haematobium* were counted and recorded for each individual separately. Of note, urine and stool samples may be sources of bacterial, parasitic and viral infections. Hence, children were asked to clean their hands with soap after producing and transferring stool and urine samples. At the end of the study, all school-aged children were treated with praziquantel at a single oral dose of 40 mg/kg [3].

### Questionnaire survey

A questionnaire survey was conducted in November 2016, addressed to those children who participated in the parasitological survey in the six villages chosen for in-depth analyses. The interviewers were well acquainted with the study setting and received prior training and specific instructions to conduct the interviews. Two different questionnaires were employed; the first questionnaire pertained to recent history of preventive chemotherapy (Additional file 1: Text S1; in French) [34], while the second questionnaire investigated schistosomiasis risk factors (Additional file 2: Text S2; in French).

The schistosomiasis risk factor questionnaire assessed demographic data, house construction materials, main source of water for drinking and cooking, socioeconomic factors, latrine access and use, defecation behaviours, hygiene, contact to open surface water and other water-related activities. For the present study, we defined stagnant water as a freshwater that does not flow (e.g. pond, lake or backwater). The history of preventive chemotherapy questionnaire has been described elsewhere [34]. In brief, the main focus was on children’s participation at the last school-based treatment campaign in late 2015 and knowledge on schistosomiasis and praziquantel treatment. In case children did not take the medication, they were asked about the underlying reasons.

### Malacological survey

In December 2016, a malacological survey was conducted for the identification of schistosomiasis intermediate host snails. In each of the six target villages, all human-water contact sites located within a buffer of 4 km from the village centre were identified after discussion with local authorities. These human-water contact sites were grouped into ponds, small dams, swamps, rivers, lakes, paddy fields and streams. Snail sampling was conducted by two experienced field workers. Snails were collected during 15 min at each site by hand using soft tweezers. In addition, a long-handed kitchen scoop (1.5 m pole; 0.8–1.2 mm mesh size) was used to reach under deeper vegetation and to sample where access was difficult. Collected snails were identified to the genus level and, whenever possible, to the species level [35]. Potential intermediate host snails of schistosomiasis (i.e. *Biomphalaria* and *Bulinus*) were placed in screw top plastic containers with freshwater from the site and transferred to a nearby laboratory. The remaining snails were put back into the water body. Human-water contact sites were georeferenced using a hand-held phone (Hot 2 X510; Infinix, Guangzhou, People’s Republic of China). Potential schistosomiasis intermediate host snails were reared in the laboratory and subjected to cercarial shedding at days 1, 15 and 30 after collection. The snails were exposed to artificial light from 11:00 to 14:00 h.

### Snail habitat data

Human-water contact sites were characterised (e.g. accessibility, presence of water during the year, estimated canopy coverage, vegetation and substratum). Water flow, level and depth were also registered. The presence of domestic animals (e.g. cows, horses, goats, dogs, sheep, pigs and donkeys) and wild animals (e.g. monkey and birds) was documented through direct observations, by recording footprints and information given by local residents. Water contact activities such as laundry, swimming, playing, water collection, rice growing, crossing river, fishing, open defecation, washing bicycles and cars were determined by direct observations. Additionally, key stakeholders (e.g. heads of households and mothers of young children) were asked whether the aforementioned water contact activities were practiced in their location. Water samples were collected in 1.5 l plastic bottles and transferred to a laboratory in Abidjan for determining physico-chemical parameters: total dissolved solids (mg/l), conductivity (μS/cm) and pH, recorded using a portable multimeter (Hanna Instruments, Woonsocket, USA).

### Statistical analysis

Data were entered into Microsoft Excel 2010 (Microsoft Corporation; Redmond, USA) and cross-checked with EpiInfo version 3.5.4 (Centers for Disease Control and Prevention; Atlanta, USA). Statistical analyses were performed with STATA version 13.1 (Stata Corporation; College Station, USA). Age was stratified into two groups: 9–10 and 11–12 years. Intensities of *Schistosoma* infection were classified according to WHO guidelines [36]. In brief, intensity of *S. mansoni* infection was categorised into light (1–99 eggs per gram of stool (EPG)), moderate (100–399 EPG) and heavy (≥ 400 EPG). Intensity of *S. haematobium* infection was grouped into light (1–49 eggs/10 ml of urine) and heavy (≥ 50 eggs/10 ml of urine). All statistical analyses involving comparisons or associations across schools took into account the clustered data structure treating schools as primary sampling units. Stata’s survey methodology was used for this purpose. Proportions of outcomes were compared according to sex, age group and whether or not a school was considered PHS. Statistical significance was defined at the 5% level. Logistic regression models were used to examine the relationship between the odds of *S*. *mansoni* and potential risk factors.

## Results

### Prevalence of *Schistosoma* infection

Table 1 summarises the prevalence of *Schistosoma* infection, stratified by sex, age group and school. Schistosome eggs were detected in the stool and urine of 33 of the 274 children surveyed (12.0%) in October 2016. The prevalence of *S. mansoni* and *S. haematobium* were 9.5% and 2.6%, respectively. None of the children were concurrently infected with both *S. mansoni* and *S. haematobium*. From the initial 75 SCORE schools, six were included in the present study. In Gbadrou, Mona, Ziondrou and Zoukougbeu, *S. mansoni* prevalence failed to decrease by at least 35% and/or the intensity of infection failed to decrease by at least 50% when comparing the baseline situation in 2012 with the endline situation in 2016 after multiple rounds of treatment (Table 2). Hence, these four schools were considered PHS in the present analysis. The remaining two schools (Tobly Bangolo and Zê) achieved a *S. mansoni* prevalence reduction by at least 35% and/or the intensity of infection declined by at least 50%. Subsequently, these two villages were considered low-prevalence schools. In general, prevalence and intensity of *S. mansoni* infection decreased meaningfully in schools where children received four rounds of praziquantel treatment compared with schools where only two treatments were performed.

**Table 1:** Prevalence of *Schistosoma* infection in children aged 9–12 years in the six schools of a large SCORE study in western Côte d’Ivoire in October 2016

Variable	Children examined	*S. mansoni*		*S. haematobium*	
	*n*	%	95% CI	%	95% CI
Sex					
Boys	150	10.1	5.5–17.1	1.3	0.2–4.8
Girls	124	8.8	2.2–21.4	4.0	1.3–9.1
Age group^a^ (years)					
9–10	166	9.0	4.3–16.6	0.0	0.0–1.8
11–12	108	10.2	2.4–23.2	6.5	1.0–19.2
Schools					
Persistent hotspot school					
Gbadrou	44	13.6	5.2–27.4	0.0	0.0–6.6
Mona	46	4.3	0.5–14.8	0.0	0.0–6.3
Ziondrou	44	22.7	11.5–37.8	4.5	0.6–15.5
Zoukougbeu	49	4.1	0.5–14.0	8.2	0.2–16.1
Total	183	10.9	6.4–15.5	3.3	1.2–7.0
Low-prevalence school					
Tobly Bangolo	46	8.7	2.4–20.8	0.0	–
Zê	45	4.4	0.5–15.1	2.2	0.1–11.8
Total	91	6.6	1.4–11.8	1.1	0.0–6.0

^a^Significant difference between the two age groups in the prevalence of *S. haematobium* infection (*P* < 0.001)

**Table 2:** Prevalence and intensity of *S. mansoni* infection in children aged 9–12 years at baseline (2012) and endline (2016) in six selected schools in western Côte d’Ivoire

School	Study arm	No. of treatment rounds	Baseline (2012)			Relative change (comparing endline with baseline)		School category
			Prevalence	Arithmetic mean egg counts (EPG)	Treatment coverage (%)	Prevalence (%)	Intensity (%)	
Gbadrou	C	2	19.0	114.9	77	− 37	104	PHS
Mona	A	4	14.0	65.8	109	− 71	− 15	PHS
Ziondrou	B	2	32.7	81.0	79	− 33	− 6	PHS
Zoukougbeu	B	2	15.2	22.6	74	− 60	118	PHS
Tobly Bangolo	A	4	26.0	33.0	71	− 69	− 70	Low-prevalence
Zê	A	4	27.0	71.4	96	− 85	− 50	Low-prevalence

*Abbreviations*: PHS, persistent hotspot

The average *S. mansoni* prevalence in the four PHS was 10.9% (95% CI: 6.8–16.4%), while it was 6.6% (95% CI: 2.5–13.8%) in the two low-prevalence schools. The highest *S. mansoni* infection prevalence was found in Ziondrou (22.7%, 95% CI: 11.5–37.8%). The arithmetic mean egg count among *S. mansoni*-positive individuals was 123.8 EPG in the four PHS schools and 18.7 EPG in the two low-prevalence schools. *Schistosoma mansoni* prevalence was significantly different between schools (*χ*^2^ = 14.3, *df* = 5, *P* = 0.014). Infections with *S. mansoni* were mainly light (80.8%), while moderate (15.4%) and heavy (3.9%) infections were less common. The two age groups did not differ significantly in the *S. mansoni* prevalence (*χ*^2^ = 0.1, *df* = 1, *P* = 0.751).

The average *S. haematobium* prevalence was 3.3% (95% CI: 1.2–7.0%) in the four PHS schools and 1.1% (95% CI: 0.03–6.0%) in the two low-prevalence schools. The highest prevalence of *S. haematobium* was found in Zoukougbeu (8.2%, 95% CI: 0.2–16.1%). Among *S. haematobium*-positive children in the four PHS schools, the intensity of the infection was 16.7 eggs/10 ml of urine (95% CI: 0.3–33.0 eggs/10 ml of urine). All *S. haematobium* infections were of light intensity (< 50 eggs/10 ml of urine). *Schistosoma haematobium* prevalence differed significantly between the two age groups with children aged 11–12 years at higher odds of infection (*χ*^2^ = 11.0, *df* = 1, *P* = 0.001).

### Demographic characteristics

Table 3 summarises the demographic characteristics of the study villages, including results from the questionnaire survey regarding the last round of preventive chemotherapy. Overall, 299 children participated in the parasitological survey. However, 25 children were absent during the questionnaire survey, resulting in 274 children with complete parasitological and questionnaire data. There were 150 (54.7%) boys. There was no significant sex difference between the two groups of schools. With regard to age, 60.6% of the children were in the younger age group (9–10 years) with no significant difference in the age groups between the PHS and low-prevalence schools (*P* = 0.805) (Table 4).

**Table 3:** Characteristics of the study population in six selected schools from a large SCORE study in western Côte d’Ivoire in November 2016

Variable	Overall		Persistent hotspot schools								Low-prevalence schools			
			Gbadrou		Mona		Ziondrou		Zoukougbeu		Tobly Bangolo		Zê	
	*n*	(%)	*n*	(%)	*n*	(%)	*n*	(%)	*n*	(%)	*n*	(%)	*n*	(%)
Sex														
Boys	150	54.7	32	72.7	26	56.5	21	47.7	21	42.9	28	60.9	22	48.9
Girls	124	45.3	12	27.3	20	43.5	23	52.3	28	57.1	18	39.1	23	51.1
Age group (years)														
9–10	166	60.6	29	65.9	31	67.4	19	43.2	30	61.2	35	76.1	22	48.9
11–12	108	39.4	15	34.1	15	32.6	25	56.8	19	38.8	11	23.9	23	51.1
Mean number of persons per house	8.3		8.7		7.2		8.8		8.5		7.9		8.7	
Present during treatment														
Yes	267	97.5	44	100	45	97.8	44	100	47	96.0	43	93.5	44	97.8
No	6	2.2	0	0	1	2.2	0	0	1	2.0	3	6.5	1	2.2
Donʼt remember	1	0.3	0	0	0	0	0	0	1	2.0	0	0.0	0	0.0
Received praziquantel														
Yes	261	95.3	43	97.7	44	95.6	44	100	49	100	37	80.4	44	97.8
No	13	4.7	1	2.3	2	4.4	0	0	0	0	9	19.6	1	2.2
Received and took all praziquantel tablets														
Yes	256	93.4	42	95.5	41	89.1	44	100	49	100	36	78.3	44	97.8
No or donʼt know	18	6.6	2	4.5	5	10.9	0	0	0	0	10	21.7	1	2.2
Don’t know what praziquantel treats														
Yes	179	65.3	34	77.3	23	50.0	23	52.3	38	77.5	38	82.6	23	51.1
No	95	34.7	10	22.7	23	50.0	21	47.7	11	22.5	8	17.4	22	48.9

**Table 4:** Demographic, socioeconomic and environmental factors, stratified by two categories of school

Factor	Persistent hotspot schools		Low-prevalence schools		*P*-value^a^
	(%)	95% CI	(%)	95% CI	
Age group (11–12 years)	37.4	15.8–65.5	40.4	28.2–54.0	0.805
Sex	54.9	43.0–66.4	54.6	38.8–69.6	0.971
Received praziquantel	11.0	2.1–41.9	1.6	0.3–7.6	0.055
Drinking water is given once at school	4.4	0.6–26.5	16.4	5.6–39.5	0.005
Drinking water is given twice at school	4.4	1.6–11.8	39.3	10.6–78.1	
Houses covered by metal roofs	82.4	51.5–95.4	77.6	35.5–95.6	0.755
Walls made of bricks, stones or cement	84.6	67.3–93.6	74.3	45.8–90.8	0.338
Floors made with cement, tiles or linoleum	83.5	80.8–85.9	82.5	51.6–95.4	0.908
Praziquantel treats worm disease or stomach ache	20.9	5.6–53.8	25.7	11.3–48.4	0.714
Latrine at home	78.0	73.9–81.7	72.1	54.0–85.1	0.367
Open defecation outside school	31.9	3.1–87.3	29.0	5.6–73.6	0.919
Mobile phone	94.5	68.2–99.3	95.6	84.4–98.9	0.815
Received and took all praziquantel tablets	38.5	37.1–39.8	41.5	26.3–58.5	0.654
Open defecation	11.0	4.9–22.8	10.4	5.2–19.7	0.893
Laundry in stagnant water	11.0	3.2–31.6	14.2	3.4–43.9	0.726
Latrine	89.0	47.0–98.7	85.8	57.1–96.5	0.789
Safe water sources	76.9	64.9–85.7	72.1	58.8–82.4	0.472
Playing in stagnant water	8.8	1.9–32.6	13.1	4.3–33.7	0.591
Electricity	69.2	26.8–93.3	77.6	59.7–89.0	0.603
Bathing in stagnant water^b^	3.3	0.4–20.7	11.5	2.5–39.3	0.217
Open surface water for drinking and cooking	3.3	0.4–21.5	9.8	6.0–15.8	0.204

^a^The respective difference in prevalence between PHS and low-prevalence schools

^b^Bathing in stagnant water source such as pond, lake or backwater

*Note*: Confidence intervals and *P*-values were calculated taking into account the clustered data structure with schools as primary sampling units

Most of the children (97.5%) declared that they were present during the last treatment round and 95.3% of them had received praziquantel. Reasons for not taking the drugs were absence for at least five days (37.5%), unwillingness to be treated based on a dose pole (25.0%), feeling healthy (12.5%), feeling too sick (12.5%) or absence of a community health worker during treatment administration (12.5%). There was no statistically significant difference between PHS and low-prevalence schools in terms of praziquantel coverage (*P* = 0.055). In Ziondrou and Zoukougbeu, all the surveyed children reported having received the drugs, while in the remaining schools some children were missed out. Two-thirds of the children (65.7%) said that they did not know which disease praziquantel cured. About one out of seven children (13.5%) was of the opinion that praziquantel cured stomach diseases. Slightly more than 10% of the children reported that praziquantel cured worms (10.6%) or other non-specified diseases (10.2%). There was no association between children’s knowledge and the prevalence of *S. mansoni* (*χ*^2^ = 0.9, *df* = 3, *P* = 0.818). There was no significant difference between PHS and low-prevalence schools in the proportion of children who reported that praziquantel cured worm disease or stomach ache (*P* = 0.714).

### Socioeconomic status and WASH indicators

**Fig. 1:**
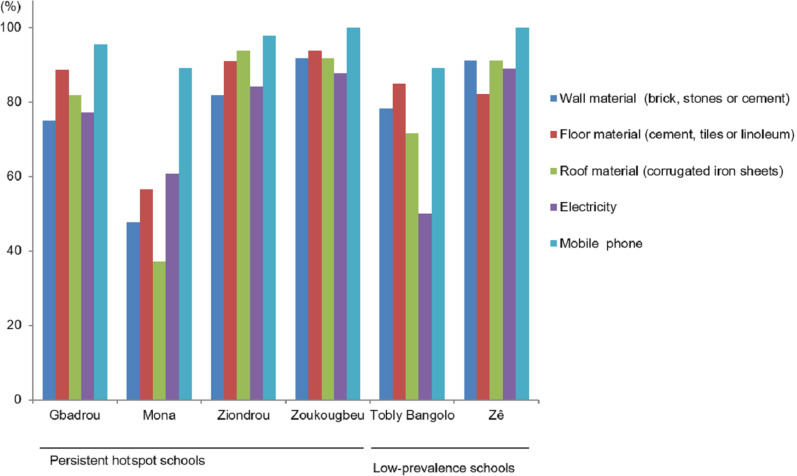
Characteristics of house building materials, access to the power grid and possession of mobile phones in six villages in western Côte d’Ivoire in 2016

Figure 1 shows the characteristics of household materials in the study villages. Most of the children in Zoukougbeu lived in houses with walls made of bricks, stones or cement (91.8%) and floors made with cement, tiles or linoleum (93.9%). The majority of children’s families had houses covered by metal roofs (91.8%). In Mona, houses were predominantly constructed with simple or natural materials for walls (52.2%) and roofs (63.0%). Three-quarters of children’s families in the study villages had electricity at home. In Tobly Bangolo, only half of the participants had access to the power grid. Mobile phones were available in the households of all children surveyed in Zoukougbeu and Zê. Electricity at home was significantly associated with households that had at least one mobile phone (*χ*^2^ = 25.6, *df* = 1, *P* < 0.001), walls made of bricks, stones or cement (*χ*^2^ = 10.4, *df* = 1, *P* = 0.001), floors made with cement, tiles or linoleum (*χ*^2^ = 5.2, *df* = 1, *P* = 0.023) and roofs made of iron sheets (*χ*^2^ = 13.3, *df* = 1, *P* < 0.001). We found a statistically significant association between households possessing at least one mobile phone and households with walls made of bricks, stones or cement (*χ*^2^ = 7.9, *df* = 1, *P* = 0.005) and roofs made of cement, tiles or linoleum (*χ*^2^ = 13.7, *df* = 1, *P* < 0.001). The odds of being infected with *S. mansoni* was higher in households with floors made with cement, tiles or linoleum compared with households having other types of floors (OR: 3.55, 95% CI: 0.56–22.60) (Table 5).

**Table 5:** Association between demographic and environmental factors and *S. mansoni* prevalence

Variable	Odds ratio	*P*-value	95% CI
Bathing in stagnant water	3.67	0.047	1.02–13.18
Latrine at home	0.25	0.013	0.10–0.64
Latrine at home or at school	1.92	0.044	1.02–3.61
Floors made with cement, tiles or linoleum	3.55	0.139	0.56–22.60

*Note*: Odds ratios were obtained from a logistic regression model including all four variables together and taking into account the clustered data structure with schools as primary sampling units

Table 6 summarises WASH indicators in the study area. There were three types of public water sources: wells, pumps and taps. In PHS schools, between 30 and 38 children per school (65.2–86.4%) mentioned that their parents collected water from safe water sources for drinking or cooking. The proportions of children who reported that their parents obtained water from safe water sources were 86.4%, 72.7%, 65.3% and 65.2% in Gbadrou, Ziondrou, Zoukougbeu and Mona, respectively. In Ziondrou (65.9%) and Gbadrou (52.3%) safe water was predominantly collected from taps. In Zoukougbeu (30.6%) and Mona (28.3%), wells were the most common source of water collection for domestic use.

**Table 6:** WASH indicators in six schools from a large SCORE study in western Côte d’Ivoire in November 2016

Variable	Overall		Persistent hotspot schools								Low-prevalence schools			
			Gbadrou		Mona		Ziondrou		Zoukougbeu		Tobly Bangolo		Zê	
	*n*	(%)	*n*	(%)	*n*	(%)	*n*	(%)	*n*	(%)	*n*	(%)	*n*	(%)
Protected well														
Yes	67	24.5	8	18.2	13	28.3	1	2.3	15	30.6	18	39.1	12	26.7
No	207	75.5	36	81.8	33	71.7	43	97.7	34	69.4	28	60.9	33	73.3
Tap water														
Yes	93	33.9	23	52.3	5	10.9	29	65.9	13	26.5	4	8.7	19	42.2
No	181	66.1	21	47.7	41	89.1	15	34.1	36	73.5	42	91.3	26	57.8
Public pumps														
Yes	42	15.3	7	15.9	12	26.1	2	4.5	4	8.2	11	23.9	6	13.3
No	232	84.7	37	84.1	34	73.9	42	95.5	45	91.8	35	76.1	39	86.7
Safe water sources														
Yes	202	73.7	38	86.4	30	65.2	32	72.7	32	65.3	33	71.7	37	82.2
No	72	26.3	6	13.6	16	34.8	12	27.3	17	34.7	13	28.3	8	17.8
Latrine at home														
Yes	203	74.1	27	61.4	32	69.6	29	65.9	44	89.8	35	76.1	36	80.0
No	71	25.9	17	38.6	14	30.4	15	34.1	5	10.2	11	23.9	9	20.0
Latrine at school														
Yes	140	51.1	2	4.5	35	76.1	36	81.8	17	34.7	9	19.6	41	91.1
No	134	48.9	42	95.5	11	23.9	8	18.2	32	65.3	37	80.4	4	9.0
Latrine														
Yes	238	86.9	28	63.6	40	87.0	44	100	45	91.8	36	78.3	45	100
No	36	13.1	16	36.4	6	13.0	0	0	4	8.2	10	21.7	0	0
Open defecation														
Yes	111	40.5	40	90.9	9	19.6	3	6.8	20	40.8	35	76.1	4	8.9
No	163	59.5	4	9.9	37	80.4	41	93.2	29	59.2	11	23.9	41	91.1

In low-prevalence schools, the number of children who reported that they used safe water sources was 37 (82.2%) and 33 (71.7%) in Zê and Tobly Bangolo, respectively. In Zê, water was mainly collected from taps (42.2%), while in Tobly Bangolo, wells were the predominant source (39.1%). The proportion of children who said that their parents collected water from safe sources was 72.1% in PHS schools and 76.9% in low-prevalence schools with no statistically significant difference (*χ*^2^ = 0.72, *df* = 1, *P* = 0.396). There was a statistically significant difference between PHS and low-prevalence schools in the proportion of children who reported receiving water for drinking (*P* = 0.005) with a higher proportion of children receiving water for drinking in low-prevalence schools.

Three-quarters of the children (74.1%) had latrines at home. Slightly less than half of the children (48.9%) had no latrine at school. In PHS, latrines were available in more than 80% of children’s houses. A high coverage of latrines at school was mainly recorded in Ziondrou (81.8%). In the low-prevalence school Zê, a high coverage of latrines both at home (80.0%) and at school (91.1%) was reported. The logistic regression analysis showed that the odds of being infected with *S. mansoni* was significantly lower among children from households with a latrine compared with household without a latrine (OR: 0.26, 95% CI: 0.11–0.63).

**Fig. 2:**
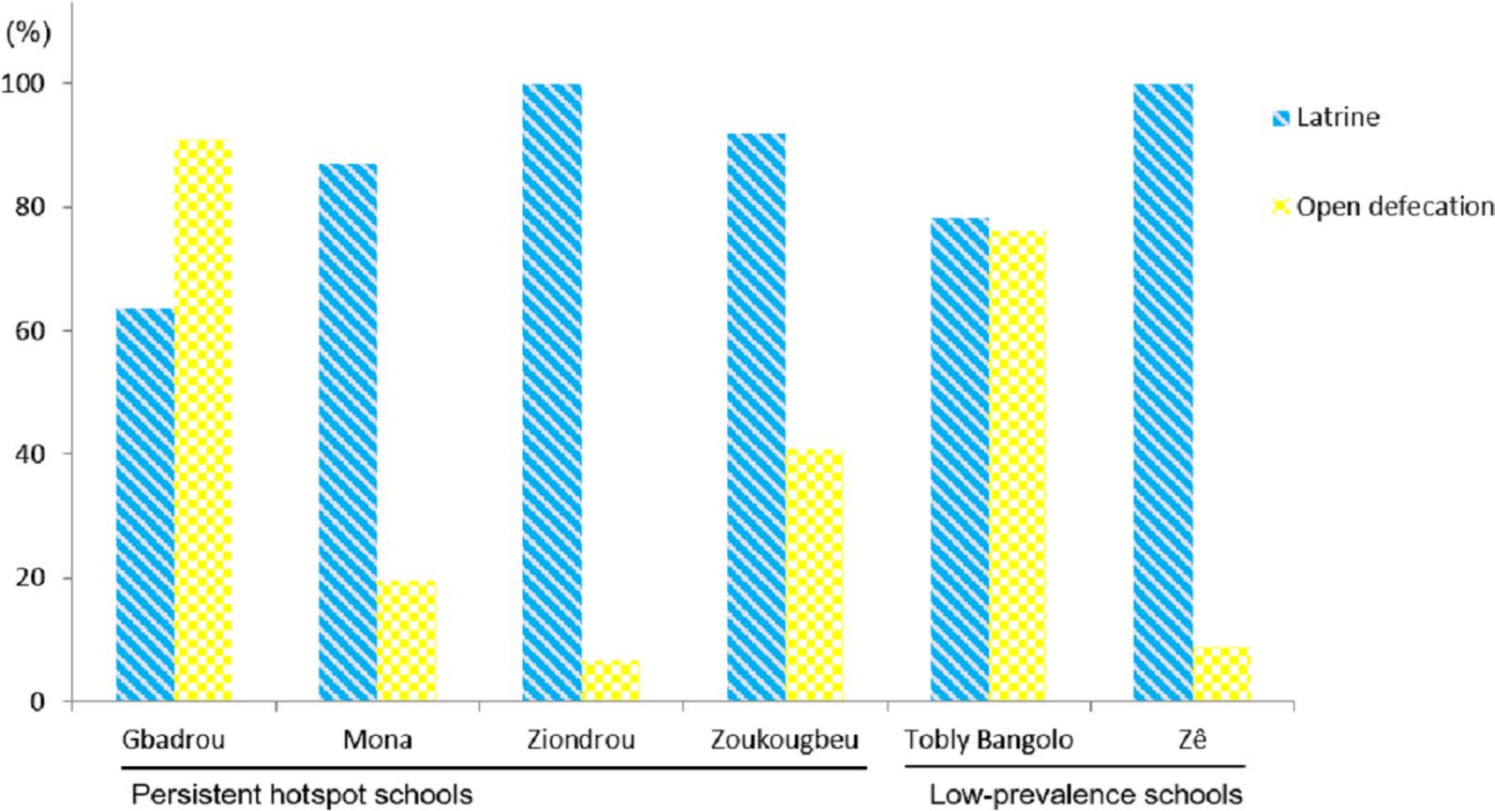
Latrine (at home and/or school) coverage and open defecation behaviour among school-aged children in six villages of western Côte d’Ivoire in 2016

Figure 2 shows latrine availability and open defecation practice, stratified by village. Open defecation was practiced in each of the six villages studied in greater depth. In general, open defecation was less practiced in villages with a high number of latrines. In PHS, less than half of the children (43.2%) from Gbadrou claimed that they never used a latrine. Most of the children in Gbadrou (95.5%) and Zoukougbeu (65.3%) did not use latrines at school. Open defecation was widely practiced in Gbadrou (90.9%). There was no significant difference between PHS and low-prevalence schools in the proportion of children practicing open defecation (*P* = 0.893).

In low-prevalence schools, the number of children who had latrines (*n* = 36) and those practicing open defecation (*n* = 35) were similar in Tobly Bangolo. Most of the children (80.4%) in the later school did not use a latrine at school while defecating at school. Open defecation was commonly practiced in Tobly Bangolo (76.1%).

### Water-related activities

Table 7 summarises the water sources for playing, laundry, bathing and collecting water for domestic or agricultural use in the study area. Most of the children (61.7%) reported that they performed the aforementioned activities in lake, pond or stream water.

**Table 7:** Water-related activities in the six schools of a large SCORE study in western Côte d’Ivoire in November 2016

Variable	Overall		Persistent hotspot schools								Low-prevalence schools				*P*-value
			Gbadrou		Mona		Ziondrou		Zoukougbeu		Tobly Bangolo		Zê		
	*n*	(%)	*n*	(%)	*n*	(%)	*n*	(%)	*n*	(%)	*n*	(%)	*n*	(%)	
Do you ever do the following activities^a^ in a lake, pond or stream?															
Yes	169	61.7	35	79.6	23	50.0	32	72.7	22	44.9	21	45.6	36	80.0	
No	105	38.3	9	20.4	23	50.0	12	27.3	27	55.1	25	54.4	9	20.0	0.028
Have you, in the past month, done any of the following activities^a^ in a lake, pond or stream?															
Yes	138	50.4	30	68.2	17	36.9	27	61.4	14	28.6	17	36.9	33	73.3	
No	136	49.6	14	31.8	29	63.1	17	38.4	35	71.4	29	63.1	12	26.7	0.402
Do you play in a dam?															
Yes	5	1.8	0	0	0	0	0	0	1	2.0	4	8.7	0	0	
No	269	98.2	44	100	46	100	44	100	48	98.0	42	91.3	45	100	0.025
Do you play in a pond?															
Yes	7	2.5	2	4.5	0	0	5	11.4	0	0	0	0	0	0	
No	267	97.5	42	95.5	46	100	39	88.6	49	100	46	100	45	100	0.059
Do you play in backwater?															
Yes	21	7.7	4	9.1	3	6.5	9	20.5	1	2.0	3	6.5	1	97.8	
No	253	92.3	40	90.9	43	93.5	35	79.5	48	98.0	43	93.5	44	2.3	0.152
Do you play in a river?															
Yes	94	34.3	23	52.3	10	21.7	9	20.5	10	79.6	11	23.9	31	68.9	
No	180	65.7	21	47.7	36	78.3	35	79.5	39	20.4	35	76.1	14	31.1	0.004
Do you wash the laundry in a dam?															
Yes	5	1.8	0	0	0	0	0	0	1	2.0	4	8.7	0	0	
No	269	98.2	44	100	46	100	44	100	48	98.0	42	91.3	45	100	0.025
Do you wash the laundry in a pond?															
Yes	7	2.6	2	4.5	0	0	5	11.4	0	0	0	0	0	0	
No	267	97.4	42	95.5	46	100	39	88.6	49	100	46	100	45	100	0.059
Laundry in backwater															
Yes	25	9.2	5	11.4	2	4.4	12	27.3	0	0	4	8.7	2	4.4	0.305
No	249	90.8	39	88.6	44	95.6	32	72.7	49	100	42	91.3	43	95.6	
Laundry in a river															
Yes	97	35.4	27	61.4	14	30.4	9	20.5	11	22.5	4	8.7	32	71.1	0.310
No	177	64.6	17	38.6	32	69.6	35	79.5	38	77.5	42	91.3	13	28.9	
Bathe in a lake															
Yes	3	1.1	0	0	0	0	0	0	1	2.0	2	4.4	0	0	0.216
No	271	98.9	44	100	46	100	44	100	48	98.0	44	95.6	45	100	
Bathe in a pond															
Yes	6	2.2	2	4.5	0	0	4	9.1	0	0	0	0	0	0	0.081
No	268	97.8	42	95.5	46	100	40	90.9	49	100	46	100	45	100	
Bathe in backwater															
Yes	16	5.8	2	4.5	1	2.2	11	25.0	1	2.0	1	2.2	0	0	0.018
No	258	94.2	42	95.5	45	97.8	33	75.0	48	98.0	45	97.8	45	100	
Bathe in a river															
Yes	69	25.2	20	45.5	7	15.2	9	20.5	8	16.3	3	6.5	22	48.9	0.538
No	205	74.8	24	54.5	39	84.8	35	79.5	41	83.7	43	93.5	23	51.1	
Collect water from a lake or dam															
Yes	3	1.1	0	0	0	0	0	0	1	2.0	2	4.4	0	0	0.212
No	270	98.9	44	100	46	100	44	100	48	97.9	44	95.6	44	100	
Collect water from a pond															
Yes	5	1.8	3	6.8	0	0	2	4.5	0	0	0	0	0	0	0.112
No	269	98.2	41	93.2	46	100	42	95.5	49	100	46	100	45	100	
Collect water from backwater															
Yes	28	10.2	5	11.4	2	4.4	15	34.1	3	6.1	2	4.4	1	2.2	0.008
No	246	89.8	39	88.6	44	95.6	29	65.9	46	93.9	44	95.6	44	97.8	
Collect water from river															
Yes	43	15.7	14	31.8	6	13.0	9	20.5	4	8.2	2	4.4	8	17.8	0.131
No	231	84.3	30	68.8	40	87.0	35	79.5	45	91.8	44	95.6	37	82.2	
Play in stagnant water															
Yes	32	11.7	6	13.6	3	6.5	13	29.5	2	4.1	7	15.2	1	2.2	0.294
No	242	88.3	38	86.4	43	93.5	31	70.5	47	95.9	39	84.8	44	97.8	
Laundry in stagnant water															
Yes	36	13.1	7	15.9	2	4.4	16	36.4	1	2.0	8	17.4	2	4.4	0.458
No	238	86.8	37	84.1	44	95.6	28	63.6	48	98.0	38	82.6	43	95.6	
Collect stagnant water for domestic or agricultural use															
Yes	34	12.4	7	15.9	2	4.4	16	36.4	4	8.2	4	8.7	1	2.2	0.014
No	240	87.6	37	84.1	42	95.6	28	63.6	45	91.8	42	91.3	44	97.8	
Bathe^b^															
Yes	24	8.8	4	9.1	1	2.2	14	31.8	2	4.1	3	6.5	0	0	0.024
No	250	91.2	40	90.9	45	97.8	30	68.2	47	95.9	43	93.5	45	100	

^a^Use stagnant water source such as pond, lake, or backwater for bathing

^b^Activities: to wash the laundry, playing, bathing and collecting water for domestic or agriculture use

According to the questionnaire survey, only 7.7% of the responders played in backwater, while in Zê most of the children (97.8%) played in this type of water. More than 60% of the children did not play in rivers, while more than half of the children did so in Zoukougbeu (79.6%) and Gbadrou (52.3%) in the PHS, and in the low-prevalence school Zê (68.9%).

Most of the children did not wash laundry in lakes or small dams, with the exception of children from Zoukougbeu. Most of the responders did not wash laundry in a river, while the majority of children did so in Gbadrou (61.4%), Mona (69.6%) and Zê (71.1%). Only 25.2% of the children bathed in the river, but in Zê, half of the children did so (50.0%).

Significant differences between the four PHS and the two low-prevalence schools were found for washing the laundry, playing, bathing and collecting water for domestic or agriculture use, playing in small dams, playing in the river, washing the laundry in small dams, bathing in backwater, collecting water from the backwater, collecting stagnant water for domestic or agricultural use and using stagnant water for bathing (*P* < 0.05). Statistically significant differences were not found for playing and washing laundry in pond (*P* = 0.059). In addition, there were no significant differences for the remaining variables of water-related activities between the four PHS and the two low-prevalence schools.

Children bathing in stagnant water were at higher odds of *S. mansoni* infection compared with children not reporting this activity (OR: 3.67, 95% CI: 1.02–13.18) (Table 3). Children from PHS schools bathed in stagnant water more frequently compared to those from low-prevalence schools (*χ*^2^ = 5.1, *df* = 1, *P* = 0.024).

### Results from the malacological survey

Table 8 summarises the results of a cross-sectional malacological survey carried out in the study area. Overall, 76 human-water contact sites were reported in the six villages. In PHS schools, the number of human-water contact sites ranged between 10 (Mona) and 19 (Gbadrou). In the two low-prevalence schools, 14 and 9 human-water contact sites were visited in Zê and Tobly Bangolo, respectively. Rivers were the most common human-water contact sites (*n* = 59). Rice paddies constituted another 20 human-water contact sites. There were 41 easily accessible human-water contact sites. Water is present throughout the year in all the visited sites. Among the surveyed human-water contact sites, 100% open canopy and 100% covered canopy were reported in 45 and 12 sites, respectively. Vegetation was found in 74 sites. The predominant substrata were mud, sand and roots, observed in 76, 70 and 69 of the sites, respectively. Domestic animals were found in 31 human-water contact sites, including pigs (31.3%), dogs (26.6%), cows (15.6%), chickens (10.9%), sheep (9.4%) and goats (6.3%). Birds were the only wild animals observed in all the surveyed human-water contact sites. The average pH was 6.2 (range: 5.0–7.1). The average total dissolved solids was 9.5 ppm (range: 4–51 ppm) and the average conductivity was 38.9 µS (range: 9–103 µS).

**Table 8:** Snail species in the six schools of a large SCORE study in western Côte d’Ivoire in December 2016

Village	Persistent hotspot schools				Low-prevalence schools		Total
	Gbadrou	Mona	Ziondrou	Zoukougbeu	Tobly Bangolo	Zê	
No. of human-water contact sites	19	10	12	12	9	14	76
Snail species							
*Biomphalaria pfeifferi*	0	77	12	0	3	0	92
*Bulinus forskalii*	2	6	3	6	4	4	25
*Bulinus globosus*	0	6	0	7	3	0	16
*Bulinus truncatus*	0	0	0	0	1	0	1
*Ferrissia eburnensis*	0	0	0	0	0	4	4
*Gyraulus costulatus*	3	13	1	6	0	0	23
*Lanistes ovum*	0	0	0	4	0	0	4
*Lymnaea natalensis*	7	54	1	21	20	0	103
*Melanoïdes tuberculata*	0	333	0	67	0	0	400
*Pila africana*	0	5	0	0	0	0	5
*Physa acuta*	0	7	0	8	0	0	15

A total of 688 snails belonging to nine genera were found, namely, *Biomphalaria*, *Bulinus*, *Ferrissia*, *Gyraulus*, *Lanistes*, *Lymnaea*, *Melanoïdes*, *Pila* and *Physa*. *Melanoïdes tuberculata* (400 specimens) was the most abundant snail species. With regard to potential schistosomiasis intermediate host snails, there were 92, 25, 16 and one specimens of *Bi. pfeifferi*, *Bulinus forskalii*, *Bu. globosus* and *Bu. truncatus*, respectively. However, none of the snails were shedding schistosome cercariae, while some snails were observed shedding xiphidiocercariae.

## Discussion

The present study was carried out in six schools in the western part of Côte d’Ivoire, where *S. mansoni* is endemic [7, 25, 26]. The study was embedded in a cluster-randomised trial, implemented from 2012 to 2016, to determine the effect of different praziquantel treatment schedules for sustaining the control of schistosomiasis mansoni in moderate endemicity settings (*S. mansoni* prevalence at baseline ranging from 10% to 24%) [7, 8, 28]. Our results, after multiple rounds of preventive chemotherapy with praziquantel, revealed considerably lower prevalence and intensity of *S. mansoni* compared to the baseline situation. Yet, there were a few PHS schools where the prevalence of *S. mansoni* did not reduce meaningfully, or even increased, despite repeated administration of praziquantel [8, 37].

Tobly Bangolo and Zê, the two low-prevalence schools included in our study, were subjected to four rounds of preventive chemotherapy with praziquantel. It is conceivable that the meaningful decrease in the prevalence and intensity of *S. mansoni* infection is due to the repeated administration of praziquantel, as part of the SCORE interventions from 2012 to 2016 [7]. However, in Mona, where schoolchildren also received four rounds of praziquantel, the intensity of *S. mansoni* infection failed to decrease by at least 50%. Continued transmission and rapid reinfection after the annual treatment campaign might explain this observation [10, 12].

We found that households in one of the PHS settings (i.e. Zoukougbeu) were economically better-off compared to the other villages. It is widely acknowledged that schistosomiasis is a disease of poverty with most cases reported from rural parts of low- and middle-income countries [22, 38]. Households with relatively higher wealth obviously have a higher ability to implement schistosomiasis preventive measures, such as WASH [21, 26]. The comparatively low prevalence of *S. mansoni* infection was probably due to high socioeconomic status.

We also found that open defecation was commonly practiced in the six study villages. As expected, open defecation was less common in villages with high numbers of latrines. Logistic regression analysis confirmed this observation; the odds of *S. mansoni* infection was lower in households with latrines compared with households without latrines, corroborating a systematic review and meta-analysis [39]. Hence, access to latrines is associated with lower odds of *S. mansoni* infection. Nevertheless, the highest *S. mansoni* prevalence was observed in Ziondrou, where the highest latrine coverage was reported. This observation points to factors other than sanitation explaining specific patterns within PHS. The idiosyncrasy in the relationship between latrine coverage and schistosomiasis prevalence has been reported before in the south-central part of Côte d’Ivoire [40]. A plausible explanation of this observation might lie in the behaviours of local residents.

Our questionnaire data showed that the proportions of households collecting water from safe water sources for domestic use were approximately the same in PHS and low-prevalence settings. In addition, no statistically significant differences were observed between PHS and low-prevalence schools with regard to the proportion of children with parents using water from safe water sources. These results indicate that the difference in schistosomiasis mansoni endemicity between PHS and low-prevalence schools was not explained by sources of water collection for drinking and cooking. Moreover, despite the availability of safe water sources, some households probably preferred using water from open surface sources. Hence, in the present study area, access to improved water supply has little impact on the transmission of schistosomiasis. Other factors that were not investigated in our study might also be at play. Our observation contradicts a major review including data from 144 studies that concluded that improved water supply and sanitation is associated with a lower prevalence and severity of schistosomiasis and other parasitic diseases [41]. One of Africa’s largest population-based cohort study carried out in KwaZulu-Natal in South Africa demonstrated that high coverage of piped water could decrease schistosomiasis significantly [42]. A parasitological survey coupled with a questionnaire and direct observation methods, assessing access to, and use of, different water sources in the district ‘des Savanes’ in northern Côte d’Ivoire revealed that access to unimproved water for any activity (including crossing rivers) was significantly associated with schistosomiasis [43]. Plausible explanations of these conflicting results in the impact of safe water might be due to differences in study populations with regard to cultural habits and water contact behaviours.

We found that children bathing in stagnant water such as lakes, ponds, backwaters or dams were at high risk of *S. mansoni* infection. Lakes, ponds, backwaters and small dams offer suitable conditions for the development of *Bi. pfeifferi*, the intermediate host snail of *S. mansoni* [44]. Hence, human-water contact through bathing in stagnant water exposes children to the risk of schistosomiasis transmission. Children from PHS settings practiced bathing in stagnant water more frequently than children from low-prevalence schools. This suggests that children in PHS schools maintained schistosomiasis mansoni transmission through bathing in stagnant water, which might explain the apparent failure of praziquantel treatment in the four PHS schools, where cured children may have been rapidly re-infected after praziquantel administration [10, 12, 45].

Data from our questionnaire survey revealed low awareness and knowledge of schistosomiasis. Similar results were reported from communities in northern Senegal after several years of health education [35] and in high schistosomiasis prevalence settings in western Kenya [36]. Lack of knowledge pertaining to schistosomiasis might be associated with low rates of health-seeking and low compliance with preventive chemotherapy [46].

Malacological data confirmed the presence of *Bi. pfeifferi*, *Bu. globosus* and *Bu. truncatus* in western Côte d’Ivoire, corroborating previous reports [27]. A unique feature of our research was the observation of *Bu. forskalii*, an intermediate host snail of *S. haematobium*. However, none of the snails subjected to shedding released schistosome cercariae. Similar results were reported from a 3-year study assessing the dynamics of freshwater snails and schistosomiasis prevalence in schoolchildren during the construction and operation of a multipurpose dam in the central part of Côte d’Ivoire [47]. In contrast, a previous study conducted from 1986 to 1989 in two villages in western Côte d’Ivoire found *Bi. pfeifferi* infection rates ranging from 3.8% to 12.7% [48]. The absence of snails shedding schistosome cercariae should be interpreted with caution, as we only pursued a single malacological survey. The presence of *Lymnaea* snails shedding xiphidiocercariae suggests that fascioliasis might be transmitted to human and domestic ruminants, such as cows, sheep and goats.

Our study has several limitations. First, a recent analysis using a dataset from Tanzania showed that PHS settings can be defined based on four different approaches, including prevalence and/or intensity of *Schistosoma* infection. The Tanzania study revealed that the same dataset yielded different numbers of PHS depending on the approach used to define them [49]. Hence, a PHS school can become a low-prevalence school and *vice versa* depending on the definition of PHS. We employed the most stringent definition of a PHS, namely that a village that did not decrease in *S. mansoni* prevalence by at least 35% and/or failed to reduce in intensity by at least 50% comparing the baseline with year 5 testing. The second shortcoming pertains to limited malacological data. Indeed, only a single malacological survey was conducted during the dry season. Previous work in the western part of Côte d’Ivoire showed that intermediate host snails for schistosomiasis are more abundant during the rainy season and at least four malacological surveys should be conducted within a 12-month period to obtain meaningful data. However, in view of financial and human resource constraints, we were unable to conduct repeated malacological surveys. Yet, human-water contact sites in close proximity to the six study villages were characterised and georeferenced, and hence, these sites could be surveyed longitudinally in the future. A third shortcoming of the study pertains to the diagnostic approach. We collected two stool samples and a single urine sample, and hence, the true prevalence of infection was underestimated [50]. Fourthly, we only included children aged 9–12 years, which might have biased the questionnaire results. Indeed, 9- to 12-year-old children might not participate in household chores as their older counterparts. Hence, caution should be taken when interpreting our findings. However, the selection of children in this age range is in line with SCORE protocols and facilitated parasitological and questionnaire surveys because children in this age are easily found at school. Older children are more ashamed and thus less likely to participate in stool and urine collection.

## Conclusions

Bathing in stagnant water bodies and availability of latrines were the main factors characterising PHS of *S. mansoni* in the western part of Côte d’Ivoire. School-aged children become infected with *S. mansoni* primarily while bathing in open freshwater bodies, such as ponds, lakes and backwater. The availability and use of latrines at home decreased the risk of *S. mansoni* infection. Although *S. mansoni* is highly endemic in western Côte d’Ivoire, children had relatively little knowledge on schistosomiasis and they commonly practiced open defecation. Potential schistosomiasis intermediate host snails were present in open surface water bodies. Hence, an integrated approach for schistosomiasis control is warranted that includes information, education and communication to initiate behaviour change, provision and sensitization on the use of latrines and snail control, in order to complement preventive chemotherapy with praziquantel.

## Supplementary information

**Additional file 1: Text S1.** Questionnaire pertaining to preventive chemotherapy (in French).

**Additional file 2: Text S2.** Questionnaire pertaining to schistosomiasis risk factors (in French).

## Data Availability

Data supporting the conclusions of this article are included within the article and its additional files. Data generated or analysed during the present study are not publicly available, but are available from the corresponding author upon reasonable request.

## Abbreviations

CI: confidence interval
EPG: eggs per gram of stool
PHS: persistent hotspot
OR: odds ratio
SCORE: Schistosomiasis Consortium for Operational Research and Evaluation
WASH: water, sanitation and hygiene
WHO: World Health Organization
