# Supplementary material for: Characteristics of persistent hotspots of Schistosoma mansoni in western Côte d’Ivoire
Source: Parasit Vectors. 2020 Jul 2;13:337. doi: 10.1186/s13071-020-04188-x (PMC7333430; doi:10.1186/s13071-020-04188-x)
Supplement: Supplementary file 1 — Additional file 1: Text S1. Questionnaire pertaining to preventive chemotherapy (in French). [file 13071_2020_4188_MOESM1_ESM.docx]

Région: |__________________| Localité: |_______________| Code No: |___|__|__|__|__|__|__|__|

Nom de l’enquêteur: __________________ Date de l’enquête (jj/ mm/aa): |__|__|/|__|__|/2016

| **Démographie:** | | | | | | |
| --- | --- | --- | --- | --- | --- | --- |
|  | 1. | Nom: __________________________________________ | | |  |  |
|  | 2. | Sexe (M/F): _________ | | |  |  |
|  | 3. | Âge: ________ans | | |  |  |
|  | 4 | Class: ________, ou analphabet: __ | | |  |  |
|  | 5. | Nom du village: __________________________________________ | | | | |
| **Localité de la distribution du praziquantel:** | | | | | | |
|  | 6. | Etiez-vous présent pendant la période de distribution du médicament pour la lutte contre les vers en Juin 2016? ___Oui ___Non ___Je ne sais pas. | | | | |
|  | 7. | Avez-vous reçu un traitement contre les vers à la maison en Juin 2016? ___Oui ___Non ___Je ne sais pas | | | | |
|  | 8. | Avez-vous reçu un traitement contre les vers ailleurs en Juin 2016? ___Oui ___Non ___Je ne sais pas. | | | | |
|  | 8a | Si oui, où? _____________________________________________________ | | | | |
|  | 9. | Avez-vous pris (avalé) tout le médicament reçu à la maison ou ailleurs? ___Oui ___Non ___Je ne sais pas. | | | | |
| **Praziquantel; un médicament très gros, sent et a un goût amer (Montrer une photo du praziquantel)** | | | | | | |
|  | 10. | Avez-vous reçu ce médicament? ___Oui ___Non ___Je ne sais pas. | | | | |
|  | 11. | Avez-vous été mesuré pour connaitre votre taille avant de prendre ce médicament? ___Oui ___Non ___Je ne sais pas. | | | | |
|  | 12. | Combien de comprimé de ce médicament avez-vous reçu? ___comprimé(s) ___Aucun ___Je ne sais pas. | | | | |
|  | 13. | Combien de comprimé avez-vous avalé? ___comprimé(s) ___Aucun ___Je ne sais pas. | | | | |
|  | 14. | Comment avez-vous avalé les comprimés? ___tous ensemble ___matin & soir du même jour ___sur plusieurs jours ___Je ne sais pas. | | | | |
| 15. Pourquoi n’avez-vous pas avalé le médicament? | | | 1 | En bonne santé | | |
|  |  |  | 2 | Je l’ai gardé pour l’utiliser quand je serai malade. | | |
|  |  |  | 3 | J’étais trop jeune | | |
|  |  |  | 4 | J’étais trop âgé | | |
|  |  |  | 5 | J’étais trop malade | | |
|  |  |  | 6 | J’étais enceinte | | |
|  |  |  | 7 | J’étais allaitante. | | |
|  |  |  | 8 | J’étais occupé/J’étais au champ. | | |
|  |  |  | 9 | J’étais absent pour moins de 5 jours. | | |
|  |  |  | 10 | J’étais absent pour plus de 5 jours. | | |
|  |  |  | 11 | Il n’y avait pas de distributeur de médicament. | | |
|  |  |  | 12 | Je n’aimais pas le distributeur de médicament. | | |
|  |  |  | 13 | Le médicament était fini. | | |
|  |  |  | 14 | Je n’étais pas informé de ce programme de distribution de médicament. | | |
|  |  |  | 15 | Mauvaise odeur/mauvais goût | | |
|  |  |  | 16 | Je n'avais pas confiance à la mesure de la hauteur (avec la toise). | | |
|  |  |  | 17 | Il n’y avait pas de nourriture disponible | | |
|  |  |  | 18 | J’étais en jeûn. | | |
|  |  |  | 19 | Religion | | |
|  |  |  | 20 | Ce médicament n’est pas efficace | | |
|  |  |  | 21 | J’avais peur des effets secondaires du médicament | | |
|  |  |  | 22 | Je n’aime pas (pas de raison spécifique) | | |
|  | 16. | Autres raison de la non-utilisation de ce médicament: _________________________________ | 23 | Je ne sais pas | | |
| 17. Ce médicament sert à traiter quelle maladie? ____________________________________ ___Je ne sais pas | | | | | | |
| 18. Echantillon de selle fourni: ___Oui ___Non  19. Echantillon d’urine fourni: ___Oui ___Non | | | | | | |
