# Supplementary material for: Characteristics of persistent hotspots of Schistosoma mansoni in western Côte d’Ivoire
Source: Parasit Vectors. 2020 Jul 2;13:337. doi: 10.1186/s13071-020-04188-x (PMC7333430; doi:10.1186/s13071-020-04188-x)
Supplement: Supplementary file 2 — Additional file 2: Text S2. Questionnaire pertaining to schistosomiasis risk factors (in French). [file 13071_2020_4188_MOESM2_ESM.docx]

**PROJET SCORE-HOT-SPOT: QUESTIONNAIRE ÉLÈVE**

**00 Nom de l’école: ___________________________________**

**01 Numéro d’identification de l’école: ____ ____ - ____ ____ ____**

**02 Date (jj/mm/aaaa) :** |____|____|/|____|____|/|____|____|____|____|

**03 Nom de l’enquêteur: ____ ____**

| **1.**  **CARACTERISTIQUES DEMOGRAPHIQUES DES ÉLÈVES** | | | |
| --- | --- | --- | --- |
| **1.0** | *Numéro d’identification de l’élève* | [___ ___ ___] | |
| **1.1** | *Sexe* | 0. Garçon | 1. Fille |
| **1.2** | Tu es dans quelle classe? | 1 2 3 4 5 6 | |
| **1.3** | Quel âge as-tu? | \|____\| | |
| **1.4** | Tu viens de quel village? |  | |

| **2. INFORMATION MENAGE** | | | | | |
| --- | --- | --- | --- | --- | --- |
| **2.0** | Combien de personnes vivent chez vous à la maison? | \|____\| personnes | | | |
| **2.1** | Combien de frères et de sœurs avez-vous? | \|____\| | | | |
| **2.2** | Votre maison a quel type de mur? *(lire les options à haute voix et choisir une seule réponse)* | 1. Pas de mur 2. Canne/Palme/en bois 3. Argile ou boue 4. Bambou 5. Cailloux ou brique or ciment 6. Bois 7. Autres 8. Je ne sais pas | | | |
| **2.3** | Votre maison a quel type de plancher? (lire les options à voix haute et choisissez une seule réponse) | 1. Terre ou sable 2. Crotte 3. Palme ou bambou 4. Planches en bois 5. Ciment ou tuiles or linoléum 6. Autres 7. Je ne sais pas | | | |
| **2.4** | Votre maison a quel type de toit? (lire les options à voix haute et choisissez une seule réponse) | 1. Pas de toit 2. Paille 3. Bambou 4. Bois 5. Toile 6. Ciment or tuiles 7. Autres 8. Je ne sais pas | | | |
| **2.5** | Dans votre maison, y a t-il les éléments suivants? (lire les options à voix haute et de remplir avec 1 = oui et 0 = non) | Electricité: [____]  Téléphone mobile: [____] | | | |
| **2.6** | Quelle est la principale source d'eau de boisson ou de cuisine dans votre maison? (**Ne pas** lire à haute voix les options et choisissez une seule réponse) | 1. Eau de surface (rivière, lac, barrage, ruisseau, canal d’irrigation, etc) 2. Puits non protégé 3. Source non protégée 4. Puits protégé 5. Source protégée 6. Forage 7. Collection d’eau de pluie 8. Eau de robinet à la maison 9. Eau de robinet dans la cour de la maison 10. Robinet ou pompe publique 11. Eau en bouteille/Fournisseur d’eau**/** Chariot avec un petit réservoir/tambour/camion-citerne   12. Autre | | | |
| **2.7** | Avez-vous une latrine à la maison? | 0. Non | | | 1. Oui |
| **2.8** | ***Si E614=oui:*** La semaine passée, quand vous étiez à la maison et vous devriez déféquer, étiez-vous allé dans la latrine … *(lire les options à haute voix)* | 0. Jamais | 1. Parfois | | 2. Toujours |
| **2.9** | A la maison, avez-vous de l’eau pour vous nettoyer après avoir déféqué? *(lire à haute voix)* | 0. Jamais | 1. Parfois | | 2. Toujours |
| **2.10** | A la maison, avez-vous un endroit pour laver les mains avec du savon et de l’eau après avoir déféqué? *(lire les options à haute voix)* | 0. Jamais | 1. Parfois | | 2. Toujours |
| **2.11** | N’avez-vous jamais mange de la terre? | 0. Non | | 1. Oui | |
| **2.12** | *OBSERVER: l’enfant porte-il des chaussures?* | 0. Non | | 1. Oui | |

| **3. INFORMATION ECOLE** | | | | | |
| --- | --- | --- | --- | --- | --- |
| **3.0** | La dernière fois vous aviez besoin de déféquer à l’école, où l’aviez-vous fait?  *Ne pas faire de suggestions.* | 1. J’ai utilisé une latrine à l’école  2. J’ai utilisez une latrine dans une maison proche de l’école  3. Attendre jusqu’à la maison  4. Défécation à l’air libre à l’école  5. Défécation à l’air libre hors de l’école (Ex: en brousse)  6. Autre | | | |
| **3.1** | Combien de fois l'école fournir de l'eau à utiliser après la défécation? (lire à haute voix les options) | 0. Jamais | 1. Parfois | | 2. Toujours |
| **3.2** | Combien de fois l'école fournit-elle de l'eau à boire? (lire à haute voix les options) | 0. Jamais | 1. Parfois | | 2. Toujours |
| **3.3** | Avez-vous votre propre pot d'eau potable (gourde eau) à l'école? (demandez à voir) | 0. Non | | 1. Oui | |
| **3.4** | *OBSERVER:* La gourde eau porte-elle le nom de l’élève? | 0. Non | | 1. Oui | |
| **3.5** | *OBSERVER:* La gourde eau est-elle propre? | 0. Non | | 1. Oui | |
| **3.6** | Combien de fois aviez-vous apporté l'eau potable de la maison à l'école? | 0. Jamais | 1. Parfois | | 2. Toujours |
| **3.7** | A l’école, y a-ti-il un endroit pour laver les mains avec du savon et de l’eau? *(lire à haute voix)* | 0. Jamais | 1. Parfois | | 2. Toujours |
| **3.8** | A l’école, où déposez-vous vos ordures? | 1. Au coin de la salle de classe 2. Dans la poubelle 3. Sur le sol dans la classe 4. Derriere l’école 5. Poubelle principale de l’école | | | |

| **4. SCHISTOSOMIASE** | | |
| --- | --- | --- |
| **4.0** | Avez-vous une fois fait les activités suivantes dans un lac, étang, ou ruisseau? | - Laver les habits - Jouer - Se laver - Prendre de l’eau pour les activités domestiques ou agricoles |
| **4.1** | Avez-vous, le mois passé, fait les activités suivantes dans un lac, étang, ruisseau? | - Laver les habits - Jouer - Se laver - Prendre de l’eau pour les activités domestiques ou agricoles |
| **4.2** | Avez-vous joué dans l’eau? | 0. Non 1. Oui |
|  | Si oui, où? | -lac/barrage  -étang  -fleuve  -marigot  -rivière |
| **4.3** | Lavez-vous vos habits (Lessive)? | 0. Non 1. Oui |
|  | Si oui, où? | -lac/barrage  -étang  -fleuve  -marigot  -rivière |
| **4.4** | Lavez-vous en générale hors chez vous? | 0. Non 1. Oui |
|  | Si oui, où? | -lac/barrage  -étang  -fleuve  -marigot  -rivière |
| **4.5** | Prenez-vous de l’eau hors de chez vous? | 0. Non 1. Oui |
|  | Si oui, où? | -lac/barrage  -étang  -fleuve  -marigot  -rivière |
